# Simulation training for emergency teams to manage acute ischemic stroke by telemedicine

**DOI:** 10.1097/MD.0000000000003924

**Published:** 2016-06-17

**Authors:** Sébastien Richard, Gioia Mione, Claude Varoqui, Arnaud Vezain, Arielle Brunner, Serge Bracard, Marc Debouverie, Marc Braun

**Affiliations:** a Department of Neurology, Stroke Unit, University Hospital of Nancy; b Centre Universitaire d’Enseignement et de Simulation Médicale, Faculty of Medicine of Nancy; c Centre d’Investigation Clinique Plurithématique Pierre Drouin, University Hospital of Nancy; d GCS Télésanté Lorraine, Villers-lès-Nancy; e Agence Régionale de Santé, région Lorraine; f Department of Neuroradiology, University Hospital of Nancy, Nancy, France.

**Keywords:** acute cerebral infarction, emergency team, hub and spoke, intravenous rt-PA, medical simulation training, telemedicine, telestroke

## Abstract

Telemedicine contributes to initiating early intravenous recombinant tissue plasminogen activator (rt-PA) treatment for patients with acute cerebral infarction in areas without a stroke unit. However, the experience and skills of the emergency teams in the *spokes* to prepare patients and administer rt-PA treatment are ill-defined. Improving these skills could vastly improve management of acute stroke by telemedicine. We developed a medical simulation training model for emergency teams to perform intravenous rt-PA treatment in a telestroke system.

From February 2013 to May 2015, 225 learners from 6 emergency teams included in the telestroke system “*Virtuall*”—in Lorrain (northeastern France)—received a standardized medical simulation training module to perform rt-PA treatment. All learners were assessed with the same pretraining and posttraining test consisting of 52 items. The percentage of right answers was determined for every learner before and after training.

Median percentages of right answers were significantly higher in the posttraining test overall (82 ± 10 vs. 59 ± 13% pretraining; *P* < 0.001), but also in all professional subgroups: physicians (88 ± 8 vs. 67 ± 12%; *P* < 0.001), paramedical staff (80 ± 9 vs. 54 ± 12%; *P* < 0.001), nurses (80 ± 8 vs. 54 ± 12%; *P* < 0.001), and auxiliary nurses (76 ± 17 vs. 37 ± 15%; *P* = 0.002).

We describe for the first time a training model for emergency teams in a telestroke system. We demonstrate significant gain in knowledge for all groups of healthcare professionals. This simulation model could be applied in any medical simulation center and form the basis of a standardized training program of *spokes* in a telestroke system.

## Abstract 2

Supplemental Digital Content is available in the text

## 1 Introduction

The benefit of thrombolytic therapy with intravenous recombinant tissue plasminogen activator (rt-PA) to treat patients at the acute phase of ischemic stroke has been widely demonstrated since 1990s.^[1,2]^ The following challenge was to develop strategies to initiate treatment as soon as possible.^[3]^ In this setting, telemedicine constitutes a solution to improve access to expertise and treatment by expanding the reach of neurologists to persons beyond their own geographic location and shortening time to treatment initiation.^[4–6]^ For this reason, telestroke systems - based on clinical evaluation and analysis of cerebral imaging by an expert stroke center (the *hub*) via telecommunication with the patient admitted to the nearest local hospital (the *spoke*)—have been developed.^[7–9]^ However, studies show that door-to-needle time is longer in “rt-PA capable” *spokes* than in the *hubs* mainly due to delays in videoconferencing.^[10]^ While the skills and qualifications of the physicians in the *hubs* are well defined, those of the medical and paramedical teams to manage stroke patients in the *spokes* remains unclear. Most telestroke systems run various training workshops for emergency teams without formal assessment. However, a standardized training program of medical and paramedical staff in these centers would help them to deliver the most accurate information to the experts, prepare patients, and administer intravenous rt-PA treatment in a time-efficient way. Here we describe medical simulation training for *spoke* teams we developed as part of our telestroke system “*Virtuall*” in the Lorraine region (northeastern France) and provide pretraining and posttraining knowledge assessment.

## 2 Methods

### 2.1 Telestroke

Our telestroke system is composed of 1 *hub* and 6 *spokes*. The *hub* is an expert center stroke unit with neurologists and radiologists specialized in the treatment of cerebrovascular diseases, a specific on-call medical service for telemedicine available 24 h/day and 7 days/week, and the capability to perform thrombectomy. The stroke patients are admitted and managed in the emergency service of the *spokes* equipped with video and audio transmission. They are linked to the *hub* via secured telemedicine software and Internet access. Examination of cerebral magnetic resonance imaging is ensured by a teleradiology system. All physicians and nurses working in the emergency department of the *spokes* had to receive a mandatory specific training before inclusion in the telestroke system. Some centers also decided to train some voluntary auxiliary nurses and radiology technicians.

### 2.2 Training

The medical simulation training took place in the *Centre Universitaire d’Enseignement et de Simulation Médicale* of the Faculty of Medicine of Nancy (France). The program was performed during a time specifically devoted to professional training for every emergency team. Learners were supervised by at least 1 neurologist from the *hub*, a physician, and a nurse—both with experience in medical simulation training—and a technician. During the simulation, 1 or 2 physicians and 2 nurses were placed in situation. The rest of the group observed the scene in a separate room through video and audio transmission. Depending on the composition of the paramedical staff attending the session, an auxiliary nurse could replace a nurse and we could include intervention of a radiology technician. We used a manikin (SimMan, Laerdal Medical), which allows monitoring of blood pressure, electrocardiogram, blood oxygen saturation, and temperature. Learners could control pulses and perform cardiac, pulmonary examinations, and urinary and venous catheterization with blood withdrawal. Vocal interaction with the manikin was possible through a facilitator concealed behind a 1-way glass. As examination of motor functions was not possible with this dummy, we used a video of the examination of an anonymized patient who presented deficiencies in line with the scenario. Learners had access to material for catheterization and blood withdrawal, dummy rt-PA, antihypertensive drugs, and 2 syringe pumps. Another thrombolytic agent, tenecteplase, was available as a confounder. We also provided tables to calculate the rt-PA dose as well as the National Institutes of Health Stroke Scale (NIHSS) score. Learners had access to the usual laboratory analyses and cerebral imaging. The telestroke was mimicked by visual control by the expert neurologist positioned behind the 1-way glass and by phone discussion. A facilitator played the role of an auxiliary nurse and helped the learners to use the medical devices and the material of the simulation room. He or she could also intervene if the learner made mistakes or omissions, which could interfere with following parts of the scenario. Every session began with a presentation of the simulation center, the manikin, and all available devices. At the beginning of the simulation, the date, time, and location of the situation were announced to the learners. The simulation was followed by a debriefing with the neurologist and the expert physician for simulation training. During this debriefing, every learning goal was broached with the learners who also received correction and advice about their attitude during the simulation. Every learner who played a part in the scenario talked about how he or she felt during the simulation. The learners who observed the scenario could make comments about the situation and highlight what they considered to be right or wrong actions. Finally, they were invited to consider how this situation could be applied in real life, in the emergency department of their hospital.

### 2.3 Simulation scenario

The patient in the scenario is a previously self-sufficient 85-year-old man with a medical history of hypertension and arrhythmia admitted to the emergency department of a *spoke* at 8:15 am following a fall and left hemiplegia (see Table 1, Supplemental Content which precisely describes the scenario). The ambulance paramedic reports that the patient was found on his bedroom floor at 6:30 am by his wife. He gives the emergency team a bag containing the patient's usual medication with vitamin K antagonists, calcium-channel blockers, and digoxin. The patient presents motor deficiency of the left side, with only slight movement of the leg, total left sensory loss, and spatial neglect. He is unaware of his deficiency, but does not present any confusion. If the learners ask, he says that he fell after waking up on his way to the toilet. He is not able to specify the exact time of the fall or the time he spent on the floor. The NIHSS score is 16. Learners can interview the patient's wife who testifies she heard the fall at 6:30 am. They have to obtain consent to use telemedicine from the patient or his wife. Blood pressure is 192/100 mmHg and is lowered to 140/60 mmHg if the learners make the right diagnosis and treat urinary retention and/or use antihypertensive drugs. Laboratory tests show normal blood count, electrolyte values, renal function, and an international normalized ratio of 1.5. Brain magnetic resonance imaging reveals an acute right ischemic stroke on diffusion-weight imaging, not yet visible on fluid-attenuated inversion recovery imaging, with signs of occlusion of the right middle cerebral artery confirmed on 3-dimensional time-of-flight angiography. The learners then need to call the expert center to report the case concisely, and give the NIHSS score and the results of the investigations. A collegial decision as to whether to administer intravenous treatment with rt-PA is made. The patient and his wife are able to give the patient's exact weight to determine rt-PA dose. Information has to be given to the patient and his wife. Learners have to raise the point of medicalized transfer to a stroke unit (see Video S1, Supplemental Content which shows a training session). Authorization has been obtained for disclosure of all recognizable persons included in video file.

### 2.4 Training assessment

Learners were made aware that they would undergo the same pretraining and posttraining test. They were all tested with the same 10 multiple choice questions (MCQ) and 4 short-answer questions based on guidelines from the French Authorities (Haute Autorité de Santé) and the American Heart Association.^[6]^ There were 52 items in all (see Table S2, Supplemental Contentwhich describes overall questions). Questions were divided into 3 subcategories: telestroke and rt-PA treatment (4 questions and 19 items); management of patients with acute ischemic stroke (7 questions and 19 items); and the radiological features of acute cerebral infarction (3 questions and 14 items). A score was attributed for each test following a standardized correction with attribution of 1 point per right answer. Learners were informed that every MCQ included at least 1 correct option. The MCQ was marked as being wrong if the learner did not choose an option. Different professional subgroups of learners were taken account: physicians, all the paramedical staff together, nurses, a paramedical subgroup of auxiliary nurses and radiological technicians, and finally auxiliary nurses alone. Learners were also told they could rate their satisfaction and the quality of the means used in the training on a scale from 0 to 5.

### 2.5 Statistical analysis

All analyses were carried out using IBM SPSS Statistics software, version 20 (SPSS Inc., Chicago, IL, USA). The percentage of correct answers obtained in the pretraining and posttraining tests were determined for each learner. These quantitative results were described as median, mean, standard deviation, range, and quartiles. A first statistical comparison was made between the scores of the pretraining and posttraining tests obtained for all the learners and then separately in the different professional subgroups. The results and improvement in the scores were also compared for overall knowledge and in the different fields of expertise between the medical and paramedical subgroups. Data were examined for normality using Shapiro–Wilk's test. Because of the non-normal distribution of all samples, the nonparametric Wilcoxon signed-rank test was used to compare matched samples (pretraining and posttraining test scores), and Mann–Whitney *U* test to compare independent samples (test scores and improvements in the medical and paramedical subgroups). A difference was considered as significant for *P* < 0.05, very significant for *P* < 0.01, and extremely significant for *P* < 0.001.

## 3 Results

**Figure 1:**
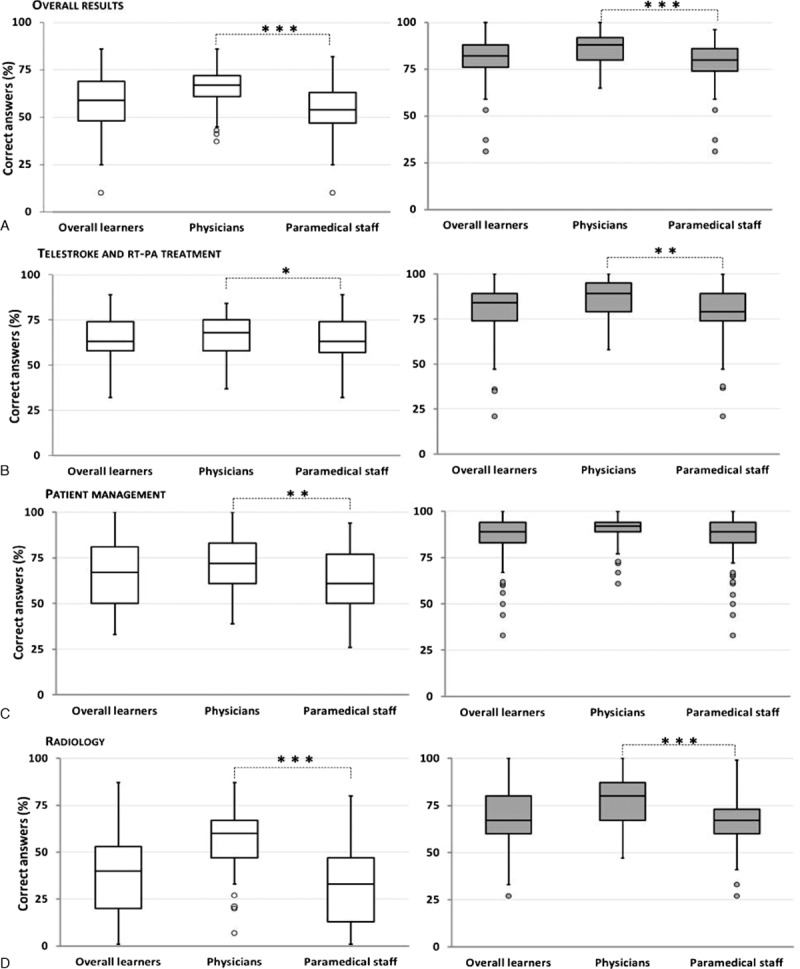
Results of overall and subcategories of pretraining and posttraining tests in medical and paramedical professional subgroups. Tukey's box-and-whisker plots, box limits: interquartile range (IQR), middle line: median, vertical lines: adjacent values (first quartile—1.5 IQR; third quartile + 1.5 IQR), dots: outliers, white boxes: pretests, gray boxes: posttests, significant difference between medical and paramedical subgroups determined with Mann–Whitney *U* test, ^∗^*P* < 0.05, ^∗∗^*P* < 0.01, ^∗∗∗^*P* < 0.001. Correct answer percentages for overall questions (A), questions about telestroke and thrombolysis (B), questions about patient management (C), and questions about neuroradiology (D).

**Figure 2:**
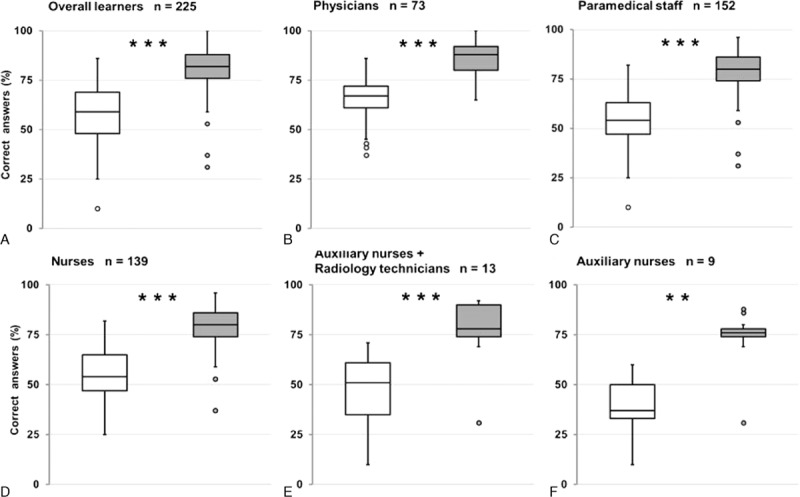
Results of pretraining and posttraining tests in overall learners and in different professional subgroups. Tukey's box-and-whisker plots, box limits: IQR, middle line: median, vertical lines: adjacent values (first quartile—1.5 IQR; third quartile + 1.5 IQR), dots: outliers, white boxes: pretests, gray boxes: posttests, significant difference between pretraining and posttraining test determined with Wilcoxon signed-rank test, ^∗∗^*P* < 0.01, ^∗∗∗^*P* < 0.001. Correct answer percentages for overall learners (A), physicians (B), paramedical staff (C), nurses (D), paramedical subgroup including auxiliary nurses and radiology technicians (E), and auxiliary nurses (F).

**Figure 3:**
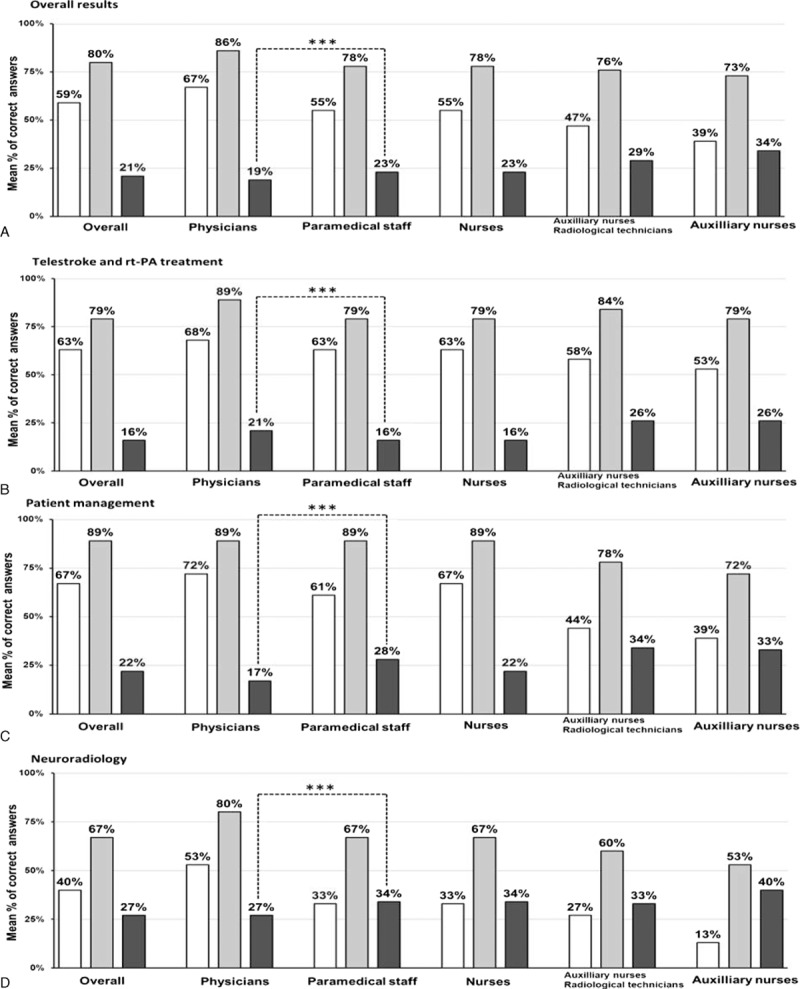
Mean percentage of correct answers and improvement for learners in overall training tests and subcategories. Pretraining tests: white histograms, posttraining tests: gray histograms, differences: black histograms, significant difference of improvement between medical and paramedical subgroups determined with Mann–Whitney *U* test, ^∗∗∗^*P* < 0.001. Mean correct answer percentages for overall questions (A), questions about telestroke and thrombolysis (B), questions about patient management (C), and questions about neuroradiology (D).

From February 2013 to May 2015, 225 learners from emergency teams including 73 physicians, 139 nurses, 9 auxiliary nurses, and 4 radiology technicians underwent the training in sixteen sessions. Pretraining test scores were significantly higher in the physician subgroup than in the paramedical subgroup (Fig. 1). Overall, the learners median percentage of correct answers significantly increased from 59 ± 13% for the pretraining test to 82 ± 10% in posttraining test (*P* < 0.001). This was also observed in the physician subgroup (67 ± 12 vs. 88 ± 8%; *P* < 0.001), paramedical staff subgroup (54 ± 12 vs. 80 ± 9%; *P* < 0.001), nurse subgroup (54 ± 12 vs. 80 ± 8%; *P* < 0.001), auxiliary nurse/radiological technician subgroup (51 ± 18 vs. 78 ± 15%; *P* < 0.001), and auxiliary nurse subgroup (37 ± 15 vs. 76 ± 17%; *P* = 0.002; Fig. 2). Posttraining test scores remained significantly higher in the medical subgroup compared with the paramedical subgroup except for knowledge about patient management where the scores were similar (Fig. 1). The mean rate of overall correct answers increased by 21% in the posttraining test, ranging from 19% in the physician subgroup up to 34% in the auxiliary nurse subgroup (Fig. 3). The improvement in scores was significantly higher (*P* < 0.001) in the paramedical subgroup than in the physician subgroup both overall and for the subcategories except for knowledge about telestroke and rt-PA treatment (Fig. 3). Feedback was excellent with more than 95% of learners rating the training 5/5 (data not shown).

## 4 Discussion

The advantages of telemedicine for administering intravenous rt-PA treatment to ischemic stroke patients are now well demonstrated. The principle is based on saving time by treating the patient at the nearest hospital and thus avoiding long transport times to a stroke unit. Telestroke systems are based on communication between the *hub* expert center and the *spoke* emergency department admitting the patient. However, studies have shown that the shorter time between stroke onset and admission is offset by a longer door-to-needle time than bedside treatment in a stroke unit.^[10]^ The main reason of this intrahospital delay is due to setting up and running the videoconferencing. This limitation has now became all the more critical as patients with occlusion of a vessel of large caliber must be transported as soon as possible from the *spoke* to a center with thrombectomy capabilities.^[11,12]^ We suggest that this time could be shortened by emergency teams being better trained to receive and prepare the patient. There are no clear guidelines about skill requirements of these medical and paramedical teams. Nevertheless, the neurologist in the *hub* expects an accurate neurological examination, an adapted therapeutic attitude concerning some key points such as blood pressure, and an early identification of factors which would contraindicate rt-PA treatment (e.g., time from stroke onset) or increased hemorrhagic risk (e.g., antithrombotic therapies). Precious time can be lost if the expert has to instruct an inadequately trained physician via videoconferencing. This formed the basis of the learning goals of our training session. Moreover, the decision to initiate rt-PA treatment is shared between the *hub* and the *spoke*. The physician at the bedside cannot be solely considered as a subordinate of the *hub* physician. This underlines the need for *spoke* physicians to acquire skills in diagnosing stroke including examination of cerebral imaging and understanding the indication for intravenous rt-PA treatment. Timely intervention of the paramedical staff is also crucial in initiating rt-PA treatment with some systematic procedures such as weighing a patient with motor deficiencies who is unable to stand, adequately administrating rt-PA and ensuring efficient monitoring.^[13]^ Thus, the *spoke* nurses have to be as skilled as their *hub* counterparts to perform this treatment. The quality of the treatment is also based on synergic action between the medical and paramedical operators. For these reasons, we train physicians and nurses together. Other healthcare professionals, like auxiliary nurses and radiology technicians, also voluntarily joined the training. This wide scope of professional qualifications did not hinder the training process with all learners expressing a high rate of satisfaction despite different learning goals. Furthermore, the learners were pleased to broach the procedures in a multidisciplinary way. Simulation training is known to improve cohesion and communication inside healthcare teams, and leadership of experienced professionals.^[14]^ Despite a previously higher level of knowledge for the physicians compared with the paramedical staff, we demonstrated a highly significant posttraining improvement in knowledge for the paramedical staff. Furthermore, the scores obtained for expertise about patient management, which represents a key point for telestroke, was similar for both professional subgroups.

To the best of our knowledge, medical simulation has not so far been used for training professionals to treat ischemic stroke. In the field of neurology, some recent studies have described this training approach to teach how to diagnose brain death, and perform surgical or technical procedures.^[15–18]^ However, it remains underutilized principally due to difficulties in making neurological examinations on a dummy. To address this limitation, we chose a scenario where the patient is able to talk to facilitate examination, and included a video to show motor deficiency. In the various applications of medical training, the management of a patient with ischemic stroke and rt-PA treatment represents the most complex, but also the most interesting. The challenge is to improve skills of an entire team in the diagnosis, management, and treatment of this critical urgent condition in a time-efficient way.^[19]^ The advantage of combining conceptual presentations with simulation training is to improve acquisition of theoretical knowledge. The debriefing after the simulation is an integral part of the training allowing correction and validation of every learning goal, each one representing a required skill to manage the stroke patient. Beyond this, the team can raise issues which could obstruct the implementation of procedures in their department. Many emergency department physicians had previously been reluctant to sign up for our telestroke system for fear of work overload. The simulation training succeeded in convincing them by demonstrating the procedures, meeting the experts, and entering into frank discussion about points of contention.

Our training model and especially the assessment method has some limitations. Application of the scenario could be improved by including the weighing equipment and a simulation of the software used in the telestroke. We were not able to assess medical and paramedical patient management separately due to the few questions in this subcategory. Furthermore, while our evaluation with pretraining and posttraining testing demonstrated an obvious gain in knowledge following the training, this might not necessarily translate the same improvement in the skills required to manage this complex situation in a real-life setting. The evaluation of our training model, as for most other studies, only reached the second level of the Kirkpatrick's hierarchy. In order to demonstrate the safety of the system, we conducted an observational study to compare rt-PA treatment initiation directly at the bedside in our stroke unit and through telemedicine in the first 2 active *spoke* centers. We did not find any significant difference for time-to-treatment and patient outcome.^[20]^ However, this first assessment is not sufficient to confer a clinical significance to our training model. The protocol has now to be implemented in other areas with rigorous evaluation to compare intrahospital times in *spoke* centers and patient outcomes before and after training. Furthermore, the gain in knowledge and operational skills of this simulation training compared to classic theoretical teaching also remains to be demonstrated. Moreover, the improvement in the posttraining test partially reflects the results of immediate knowledge after training. Even if we can consider that the skills are maintained and improved by repeating the procedures in reality, long-term evaluation should be performed.

In conclusion, our medical simulation model is a useful tool not only to train the medical and paramedical teams of *spoke* emergency departments to examine stroke patients and administer rt-PA treatment, but also to support the organization of telestroke. It can be simply applied in all medical simulation centers and could be the basis of a standardized training for medical and paramedical staff in this setting. Further studies should now try to demonstrate the clinical gain brought about by this action in terms of time to treatment and patient outcome.

## Acknowledgments

List of collaborators: Lisa Humbertjean, Ana-Maria Enea, Jean-Christophe Lacour, Gerard Audibert, Jean-Michel Kleffert, Hind Hani, Stéphanie Langard, Frédèrique Lesage, Jean-Louis Fuchs, René Anxionnat, Anne-Laure Derelle, Charlotte Barbier, Romain Tonnelet. These 13 last collaborators on behalf of the « VIRTUALL group ».

Renaud Fay as biostatistician for having reviewed Statistical Analysis and Results parts, and Felicity Neilson (Matrix Consultants) for having reviewed the English language with scientific expertise.

All these persons gave permission to be named in this section.

## Supplementary Material

Supplemental Digital Content

## Supplementary Material

Supplemental Digital Content
