# Supplementary material for: Simulation training for emergency teams to manage acute ischemic stroke by telemedicine
Source: Medicine (Baltimore). 2016 Jun 17;95(24):e3924. doi: 10.1097/MD.0000000000003924 (PMC4998489; doi:10.1097/MD.0000000000003924)
Supplement: Supplemental Digital Content [file medi-95-e3924-s001.pdf]

MEDICAL SIMULATION SCENARIO

SITUATION	FACILITATORS
**An 85-year old man admitted for a fall due to left side paralysis**<br><br>➢ **Place: admission** in a room of an emergency department with telestroke and brain imaging (MRI in our scenario)<br>➢ **Time:** 8:15 am<br>➢ **Time of stroke onset**: patient found by his wife on his bedroom floor at 6:30 am<br>➢ **Known medical history**: Cardiac arrhythmia<br>➢ **Usual medications**:<br>▪ VKA<br>▪ Calcium-channel blockers<br>▪ Digoxin<br>▪ Allopurinol<br>▪ Ginkgo biloba extract<br>➢ **Main clinical features**:<br>▪ Motor deficiency of the left side<br>▪ Loss of sensation of the left side<br>▪ Left spatial neglect<br>▪ Anosognosia<br>▪ No confusion<br>▪ Unable to give the precise time of the fall<br>➢ **Additional information**: His wife is present in the waiting room or reachable by phone	➢ **Auxiliary nurse** in the emergency room<br>➢ **A voice** for the patient/manikin<br>➢ **An ambulance paramedic**<br>➢ **The wife** if possible or her voice by phone<br>➢ **A neurologist**

Progression	Material equipment	Learning goals
**Admission of the patient**		
**The ambulance paramedic** gives the notion of<br>▪ Fall and left side paralysis, the patient was found on his bedroom floor at 6:30 am by his wife<br>▪ Medical history of cardiac arrhythmia with a bag containing usual medications<br>▪ High blood pressure measured at home (190/80 mmHg)<br>▪ Patient’s wife is in the waiting room or reachable by phone.<br><br>**The patient:**<br>▪ Unaware of his deficiency<br>▪ Not confused<br>▪ Fell while going to the toilet<br>▪ Unable to give the exact time of the fall<br><br>**Vital parameters**:<br>▪ Blood pressure: 192/100 mmHg<br>▪ Heart rate: 80 beats per minute<br>▪ Blood oxygen saturation: 97%<br>▪ Temperature: 37.2°C<br>▪ Capillary blood glucose: 6.7 mmol/L<br>▪ Electrocardiogram: atrial fibrillation	▪ Monitor with<br>- blood pressure<br>- electrocardiogram<br>- blood oxygen saturation<br>▪ Blood glucose meter<br>▪ Electronic thermometer<br>▪ 12-lead electrocardiogram<br>▪ A phone<br>▪ A plastic bag with patient’s medications	➢ To collect vital parameters<br>➢ To determine exact time of stroke onset<br>➢ To reassure the patient and to give information<br>➢ To take account of VKA treatment<br>➢ To perform electrocardiogram and to diagnose atrial fibrillation

**CLINICAL EXAMINATION**		
**On the manikin:**<br>▪ Patient unable to see anything on the left side<br>▪ Unable to feel anything on the left side<br>▪ Unaware of his deficiency<br>▪ Can give correct answers about time and place<br>▪ Moderate dysarthria and no sign of aphasia<br><br>**On the video:**<br>▪ Patient presents motor deficiency of the left side (facial, upper and lower limbs)<br><br>**NIHSS score is about 16**	▪ Table to calculate NIHSS score<br>▪ Anonymized video of a patient according to the scenario	➢ To assess orientation in time and space<br>➢ To assess motor deficit<br>➢ To assess loss of sensation<br>➢ To assess spatial neglect<br>➢ To estimate NIHSS score
**BLOOD TESTS**		
**The patient**<br>▪ Pain if the nurse learner withdraws blood and venous catheterization on the right side<br>▪ No pain if catheterization on the left side<br>▪ He wants to take his daily VKA dose.<br><br>**The auxiliary nurse**<br>▪ Gives results of laboratory tests during the session reporting no significant anomaly and an INR level of 1.5.<br>▪ If leaners did not specifically request INR, the auxiliary nurse asks them if patient is taking anticoagulant therapy because coagulation tests are abnormal.<br>▪ Asks about the daily VKA intake: "The patient's wife in the waiting room says he has not taken his medication this morning. Should we give him VKA now?"	▪ Equipment for venous catheterization and blood collection<br>▪ Normal results for complete blood count, electrolytes and renal function. Coagulation tests with INR level at 1.5	➢ To perform venous catheterization<br>➢ To request relevant laboratory analyses and INR<br>➢ To perform blood withdrawal<br>➢ To take account of INR<br>➢ To know the range of INR for rt-PA treatment

**MANAGEMENT OF BLOOD PRESSURE**		
**The patient**<br>▪ Presents urinary retention with pain during abdominal palpation<br>▪ Initial high blood pressure (192/100mmHg)<br>▪ Lowered (140/60mmHg) if learners:<br>- Perform urinary catheterization<br>- And/or initiate intravenous antihypertensive drugs	▪ Equipment for urinary catheterization<br>▪ Intravenous antihypertensive drugs<br>▪ A first syringe pump	➢ To acknowledge that blood pressure is too high for rt-PA treatment<br>➢ To diagnose urinary retention<br>➢ To treat urinary retention<br>➢ To administer antihypertensive drugs<br>➢ To know blood pressure goal for rt-PA treatment
**BRAIN IMAGING**		
Learners have to **request brain imaging**.<br>MRI has been chosen in this scenario.<br>▪ Examination can be performed with or without the expert neurologist.<br>▪ Recent right ischemic stroke present on diffusion-weighted imaging<br>▪ Not present on FLAIR imaging<br>▪ No bleeding on T2*-weighted gradient echo imaging<br>▪ Occlusion of the right middle cerebral artery on 3D TOF angiography	▪ A brain MRI with<br>- right recent ischemic stroke<br>- occlusion of the right middle cerebral artery according to the scenario<br>- diffusion-weighted imaging<br>- FLAIR imaging<br>- T2*-weighted gradient echo imaging<br>- 3D TOF angiography	➢ To ask for cerebral MRI<br>➢ To reassure the patient and to give information<br>➢ To know different MRI sequences for stroke diagnosis<br>➢ To examine MRI and form final diagnosis
**PRESENTATION OF THE PATIENT TO THE EXPERT**		
**The expert can direct the situation** if necessary for:<br>▪ Determination of exact time of stroke onset<br>▪ Estimation of NIHSS score<br>▪ Results of coagulation tests<br>▪ Management of blood pressure<br>▪ Brain imaging examination<br>▪ The choice of the drugs<br>▪ Discussion for decision of intravenous rt-PA treatment	▪ A phone	➢ To obtain consent from the patient or his relative for telemedicine<br>➢ To call the expert center<br>➢ To summarize clinical situation<br>➢ To give relevant results of paraclinical examinations<br>➢ To participate in treatment decision<br>➢ To agree with decision of treatment<br>➢ To organize medical transport to stroke unit

**RT-PA TREATMENT**		
**Determination of the patient's weight**<br>▪ with equipment if possible<br>▪ otherwise the patient or his wife are able to give a precise weight of 75 kilograms<br><br>**The auxiliary nurse**<br>▪ helps select the right thrombolytic drug if appropriate<br>▪ helps prepare and inject rt-PA if necessary	▪ Weighing equipment if possible<br>▪ Rt-PA<br>▪ Another thrombolytic agent (confounder)<br>▪ A table to calculate rt-PA dose<br>▪ Equipment to prepare and inject rt-PA<br>▪ A second syringe pump	➢ To reassure and give information to the patient and the relatives<br>➢ To inform the relatives about hemorrhagic risk due to treatment and age<br>➢ To take patient's weight into account<br>➢ To choose rt-PA over other thrombolytic therapies<br>➢ To calculate the dose of rt-PA<br>➢ To administer rt-PA correctly<br>➢ To carefully monitor clinical state and blood pressure

Table 1. Scenario of medical simulation training for emergency teams to perform intravenous recombinant tissue plasminogen activator in a telestroke system

MRI: magnetic resonance imaging, FLAIR: fluid-attenuated inversion recovery (imaging), 3D TOF: three-dimensional time-of-flight (angiography), VKA: vitamin K antagonist, INR: international normalized ratio, NIHSS: National Institutes of Health Stroke Scale, rt-PA: recombinant tissue plasminogen activator.

Questions		score	
**1**	What is, or are, the contraindication(s) for treating ischemic stroke with recombinant tissue plasminogen?		
A	A patient older than 80 years	0	1
B	Daily treatment with vitamin K antagonist	0	1
C	Time from stroke onset of more than 3 hours	0	1
D	No possibility to perform magnetic resonance imaging	0	1
E	No possibility of taking advice from neurologist	0	1
**2**	To treat a patient with ischemic stroke in a *spoke* hospital, what can be considered as expertise from the *hub* hospital?		
A	Phone advice	0	1
B	Access to clinical examination by videoconferencing	0	1
C	Access to brain imaging by teleradiology	0	1
D	Videoconferencing and teleradiology	0	1
**3**	For a 60-year old male patient with ischemic stroke due to occlusion of the right middle cerebral artery, which is or are the correct answer(s) concerning time from onset to perform intravenous recombinant tissue plasminogen activator treatment?		
A	Exclusively less than 3 hours	0	1
B	Exclusively less than 4.5 hours	0	1
C	Exclusively less than 6 hours	0	1
D	As soon as possible	0	1
**4**	Which brain magnetic resonance imaging sequence reveals ischemic stroke the earliest?		
A	Diffusion-weighted imaging	0	1
B	Fluid-attenuated inversion recovery imaging	0	1
C	T2*-weighted gradient echo imaging	0	1
D	Three-dimensional time-of-flight angiography	0	1
**5**	Which brain images can reveal occlusion of an intracranial vessel?		
A	Cerebral computed tomography without contrast	0	1
B	Cerebral computed tomography angiography	0	1
C	Diffusion-weighted resonance imaging	0	1
D	Fluid-attenuated inversion recovery resonance imaging	0	1
E	Three-dimensional time-of-flight resonance angiography	0	1
**6**	Who can decide to administer intravenous recombinant tissue plasminogen activator treatment for ischemic stroke?		
A	All physicians	0	1
B	You after this training	0	1
C	A neurologist	0	1
D	A neurologist with specialization in the cerebrovascular field	0	1
E	None of the above	0	1

**7**	Which nursing procedures are required to examine and treat a stroke patient with intravenous recombinant tissue plasminogen activator?		
A	Venous catheterization	0	1
B	Blood withdrawal for blood count and coagulation tests	0	1
C	Determining the patient's weight	0	1
D	Electrocardiogram	0	1
E	Vital parameter measurements (blood pressure, temperature, capillary glucose level)	0	1
F	Urinary catheterization	0	1
**8**	What is required to treat a patient presenting an ischemic stroke with recombinant tissue plasminogen in a hospital?		
A	Access to magnetic resonance imaging	0	1
B	Access to brain imaging	0	1
C	Physical presence of a neurologist	0	1
D	Access to neurologist's advice	0	1
E	A stroke unit inside the hospital	0	1
**9**	A right-handed 30-year old man, without any medical history is admitted because of a sudden aphasia. He has not been able to speak or understand a single word for 30 minutes.<br>What is the NIHSS score of this patient?		
	9	0	1
**10**	If this patient presents cerebral infarction, what might one see as a result(s) of cerebral computed tomography without contrast?		
A	Normal	0	1
B	Increased density in the left middle cerebral artery territory	0	1
C	Decreased density in the right middle cerebral artery territory	0	1
D	Increased density in the left middle cerebral artery	0	1
E	None of the above	0	1
**11**	Is this deficiency severe enough to justify treatment with intravenous recombinant tissue plasminogen activator if the other required conditions are met?		
A<br>B	Yes<br>No	0	1
**12**	Following brain imaging, a decision is made to treat the patient with intravenous recombinant tissue plasminogen activator. Blood pressure is 160/90 mmHg, temperature 37.2°C, venous catheterization is performed with normal blood counts, glucose level and coagulation tests. Electrocradiogram is normal.<br>What value is lacking to initiate treatment?		
	The patient's weight	0	1
**13**	When do you perform control cerebral imaging after this patient has been treated with intravenous recombinant tissue plasminogen activator?		
	24 hours	0	1

**14**	When could you give the first dose of aspirin to this patient after the end of treatment with recombinant tissue plasminogen activator?		
**A**	Immediately	0	1
**B**	One hour later	0	1
**C**	Twenty-four hours later and possibly before control cerebral imaging	0	1
**D**	Only after control cerebral imaging	0	1

Table 2. Multiple choice questions and short-answer questions used as pre- and post-training tests

NIHSS: National Institutes of Health Stroke Scale

Questions 1, 2, 6 and 8 were used to assess knowledge about telestroke and recombinant tissue plasminogen activator, questions 3, 7, 9, 11, 12, 13 and 14 for management of patient with acute ischemic stroke, and questions 4, 5 and 10 for radiological features of acute cerebral infarction.
